# Beyond the genome: a perspective on the use of DNA methylation profiles as a tool for the livestock industry

**DOI:** 10.1093/af/vfab060

**Published:** 2021-12-17

**Authors:** Shannon Clarke, Alex Caulton, Kathryn McRae, Rudiger Brauning, Christine Couldrey, Ken Dodds

**Affiliations:** 1 AgResearch, Invermay Agricultural Centre, Mosgiel, Otago, New Zealand; 2 Department of Biochemistry, University of Otago, Dunedin, Otago, New Zealand; 3 Livestock Improvement Corporation, Hamilton, New Zealand

**Keywords:** DNA methylation, epigenetic clock, epigenome, livestock, selection

**Implications**

- Advancement in sequencing technologies is expediting functionally annotated genomes in parallel to genomic selection strategies. This is important if we are to go “beyond the genome” and include epigenetic modifications in our predication of genetic merit.
- We propose that DNA methylation can be harnessed as a molecular tool in key livestock species, through the use of epigenetic clocks to investigate health, longevity, productivity and environmental adaptation. Deviation from an epigenetic clock has the immediate potential as a molecular phenotype.
- To incorporate DNA methylation data into routine genetic merit predictions, development in high throughput, robust and cost-effective assays is needed.

Globally, one of the greatest challenges to agriculture is the requirement for rapid adaptation of animals to environmental perturbation, whether in response to changes in climate, agricultural systems, or biotic stresses. Genetic adaptation, and ultimately evolution of organisms, occurs through the natural selection of advantageous mutations in DNA sequence over time. Environmental challenges from (a)biotic stress, however, require immediate biological responses, which occur through changes in gene expression. Epigenetic modifications to the genome, such as methylation, can affect gene activity without changing the underlying DNA sequence. This is an important mechanism by which gene expression is stably altered for rapid physiological response to environmental stress. Here, we provide a perspective on the use of DNA methylation profiles as a tool for the livestock industry.

The past ~20 yr have seen impressive progress in improving the rate of genetic gain in livestock using DNA variants. This has largely been implemented through 1) parentage assays to allow pedigree-based methods for estimating genetic parameters and breeding values (**BV**s) for quantitative measurements on individuals, 2) marker-assisted selection using known causative variants for single gene traits, and 3) the use of genome-wide markers (largely through not only single-nucleotide polymorphism [**SNP**] arrays and genotyping by sequencing but also copy number variants [>1 kb in length] and smaller insertions and deletions for example) in genomic selection. This, in turn, allows genomic-based estimates of variance components and BVs for polygenic traits.

In parallel, high-quality reference genomes have been assembled for key livestock species. More recently, with improved long-read sequencing technologies, an increasing number of de novo assembled, haplotype-resolved genomes are being constructed. As such, pangenomes will soon be a reality for the sector. Overlaying these assemblies is the exceptional efforts performed by the international Functional Annotation of Animal Genomes (FAANG) consortium to address the genome-to-phenome challenge; that is, how the genome and epigenome interact with the environment to determine phenotype. Again, the continued technological advances in functional genome assays (chromatin architecture and accessibility, methylation, transcriptome, and noncoding RNAs) coupled with reduced sequencing costs and increased accuracy and efficiency will enable the association of variants (SNPs and structural) at the genome level with the regulation of expression at the epigenomic level. This work will contribute toward an increased understanding of complex traits, allowing the development of more sophisticated predictions of genetic merit.

Perhaps the greatest potential of these functional assays lies in the exploration of acquired traits at a larger scale than that previously feasible. A recent review by Zhu et al. (2021) details the dynamic nature of DNA methylation, histone modifications, chromatin remodeling, and noncoding RNA, collectively known as epigenetic mechanisms. Specifically, these epigenetic mechanisms are a response to the environment and may result in fetal epigenomic reprogramming and/or transmission to future generations. This comprehensive review is timely, providing an overview of the current state of epigenetic analysis of germ cells and embryos in mammalian livestock species and the epigenetic determinants of gamete and embryo viability. This is particularly applicable to assisted reproductive technologies that are widely used not only for research purposes but also to accelerate the genetic improvement in livestock. With advances in short- and long-read nucleic acid sequencing technologies, comprehensive profiling will enable a better understanding of epigenomic regulation during gametogenesis and embryogenesis in livestock, as well as a deeper appreciation of environmental impacts on the adult epigenome and subsequent effects in later generations. Although there is some evidence for transgenerational epigenetic inheritance in livestock, large multigenerational confirmatory studies have been limited due to the costs associated with profiling the epigenome.

The historic environment is often reflected in modifications to methylation levels, with the methylome providing a fingerprint of cumulative stress exposure, conveniently packaged within any DNA sample. Combined with advances in array- and/or sequencing-based profiling methods, harnessing the methylation status has potential to be used as a high-throughput molecular predictor of health, longevity, productivity, and environmental adaptation in livestock species. It is well known from many human and animal studies that environment can influence the methylation profile, which can impact health and welfare status (Martin and Fry, 2018; Tiffon, 2018). Moreover, heritably has been estimated as 0.19 on average for DNA methylation measured at probes with no known SNPs in humans (McRae et al., 2014) and as 0.20 for global DNA methylation rate in sheep (Hazard et al., 2020). We propose that we can go beyond enhancing our understanding of gene regulation and acquired traits, and that epigenetic profiling could be incorporated into current genomic selection models as an additional layer of information to construct more sophisticated predictors of genetic merit. Epigenetics is an important, albeit largely ignored, aspect of quantitative genetics, whereby epigenetic mechanisms can account for phenotypic (both genetic and residual) variance besides that from the DNA sequence alone. Epigenetic markers could be used in plant and animal breeding schemes either as a heritable molecular phenotype in the selection of the breeding tier (e.g., as an indicator of the health and welfare of livestock, or the persistence of forage species in pasture-based farming systems) or used to estimate the epigenomic similarity between individuals to better account for their historic environments. 

Whole-genome bisulfite sequencing (**WGBS**) is still considered the gold standard assay for methylome profiling, although single-molecule sequencing of native DNA continues to be refined. Third-generation long-read sequencing platforms provided by Oxford Nanopore Technologies (**ONT**) and Pacific Biosciences not only sequence the DNA but can also detect DNA methylation through picoampere signal intensities and polymerase kinetics, respectively. However, Pacific Biosciences requires very high coverage to confidently detect 5-methylcytosine, making it economically impractical on a mammalian-sized genome. Although these methods are expensive, researchers are continuing to develop analysis pipelines, whereby lower-depth sequencing can be utilized. There is some promise for field use of ONT sequencers, as demonstrated by simulations of low-depth genotyping data for inclusion in genomic prediction for real-time selection decisions in livestock (Lamb et al., 2020). Lamb et al. (2020) propose that the current applications of ONT sequencing in livestock agriculture are at the forefront for rapid diagnostics, base modification detection such as methylation, reference genome assembly, and genomic prediction. However, third-generation technologies and WGBS remain prohibitively expensive (sequencing depth and computational power) for the large-scale animal studies required to establish and disseminate the methylome as a molecular phenotype. To successfully investigate, establish, and incorporate DNA methylation data into routine genetic merit predictions, high-throughput, robust, and cost-effective assays need to be developed. Considerations from the sampling method in the field through to DNA extraction method, molecular assay, and downstream bioinformatic and/or statistical methods need to be addressed. Realistically, to ensure industry application of epigenetic tools, labor, consumables, and computation need to be cost-effective and efficient. Reduced complexity sequencing methods such as reduced representation bisulfite sequencing, although more cost-effective, may still be a challenge for industry implementation due to the requirement for bisulfite conversion, a procedure that reduces the stability of DNA, imposing time-sensitive processing steps. Methylation-sensitive restriction enzyme sequencing methods that do not require this additional treatment may, therefore, have more utility. Downstream analysis methods that incorporate low-depth sequencing efforts would likely still be required to meet a price point for industry uptake.

In addition to sequencing-based methods, attention should also be given to the use of array-based platforms. Establishing a livestock methylation array similar to the human arrays would expedite epigenome-wide association studies (**EWAS**) studies, as reported by the thousands of environmental impact studies on human health (disease and mental) (Li et al., 2019). Up-front development of these arrays is costly, and the purchase of substantial volumes is required to reduce the per-sample price. Furthermore, the predetermined methylation sites selected for the array remain a “fixed set” until the next iteration of development and update is completed. With the continued decline in sequencing costs, reduced complexity sequencing methods for EWAS show the greatest potential for investigating the methylome and acquired traits.

Methylation profiling shows potential for application within the livestock industry. However, when establishing a cost-efficient approach to using the methylome as a predictor trait, consideration will need to be given to regions captured by reduced-representation or array-based methods. Estimates of genetic merit using DNA variants are based on linkage disequilibrium between the markers and the causative mutation, but correlations with nearby methylation signals may not be so easily modeled due to the dynamic nature of the epigenomic landscape. Studies using reduced representational methylation profiling approaches, via either sequencing- or array-based capture, will need to proceed with this consideration in mind, at least for the short term until affordable whole-genome assays are a reality. Although current arrays have been developed around specific traits, they may also be useful for predicting other traits of interest. However, additional genomic regions will likely provide greater predictive power for a particular breeding goal. Nevertheless, valuable gains can be made using a predefined panel or reduced-representation methods to establish tools for immediate implementation in large-scale association studies, while, in parallel, discovery research should continue with higher genome specificity to improve predictions further.

The now well-established phenomenon of epigenetic clocks is a prime example to demonstrate the utility of DNA methylation as an indicator of environmental impacts. The review by Horvath and Raj (2018) discusses DNA methylation-based biomarkers and the epigenetic clock theory of aging. Deviation from these clocks can indicate slowed or accelerated human aging in response to environmental stimuli. These studies have now been extended to other mammalian species. For instance, Lu et al. (2021, preprint) have constructed three mammalian epigenetic clocks from a large-scale metanalysis of methylation data profiled using the recently released mammalian methylation array (HorvathMammalMethyl40). The clocks show similarly high accuracies (*r* > 0.96) at predicting age across all 142 mammalian species and 57 tissue types assayed in the study, using a single mathematical model. This provides great potential for the use of an epigenetic clock for livestock. Establishing an accurate biomarker of age would be of use in farming systems where it is not possible to record birth date with certainty. In such circumstances, age cannot be reliably included in genetic prediction models, which results in reduced accuracy of genomic BVs. Moreover, we envision that the applications of a livestock clock could extend well beyond the scope of chronological age estimates. Many independent studies have demonstrated that a deviation between true (chronological) age and clock-derived biological age is indicative of past and/or present health status, including stress. A hypothetical example of its use is shown in Figure 1. Deviation from the clock could be used as a predictor of biological age, which in turn could be incorporated into animal selection decisions. More specifically, early selection of animals with a “reduced biological age” could result in animals with improved longevity through resilience to stressors such as a changing climate. Robust animals that are able to withstand adversity and recover from extreme, e.g., weather events display a resilience phenotype. Epigenetically younger animals may have a preference for retainment in the flock/herd as a reflection of greater resilience to environmental stressors (Figure 1A). Alternatively, incorporation of the “biological age” in genetic evaluation, to adjust an individual’s performance, may enable better selection decisions (Figure 1B). Furthermore, Li et al. (2021, preprint) have shown that maximum lifespan, the genetic limit of longevity in an ideal environment, can also be predicted via methylation-based models. There is, therefore, untapped potential to use livestock clocks in breeding programs as a predictor for age-related production traits.

**Figure 1.**
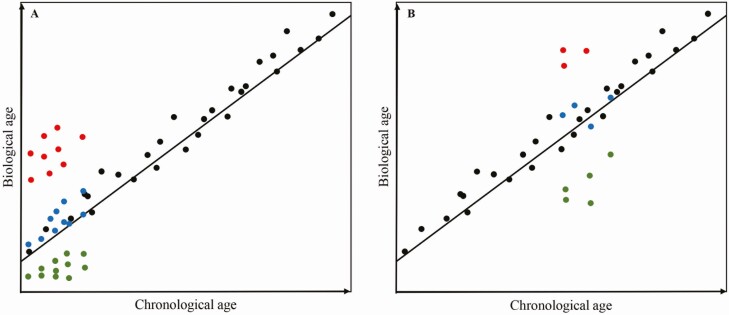
Depiction of the use of an epigenetic livestock clock in animal breeding. The black line represents the epigenetic clock established from the training animals (black dots). Animals with a younger biological age (green) compared with those that are estimated to have an older biological age (red) than their true chronological age. Those in blue have an estimated biological age that does not deviate from their true chronological age. In (A), the livestock epigenetic clock is used for early-in-life selection of animals, whereby those estimated to have a younger biological age may be selected with assumption of improved longevity, or the “biological age” could be included in genetic evaluation, to adjust an individual’s performance. In (B), the livestock epigenetic clock is used to measure historical stress, for health and welfare monitoring of animals. .

Breeding schemes within the livestock sector can likely be transformed if industry applicable molecular phenotypes, such as DNA methylation profiles, can be established and utilized to accelerate the physiological response of livestock to environmental pressures. Furthermore, this could be extended to plants, resulting in selection of animals and plants on farm with superior adaptation to disease, stress, and changing environments.

## About the Authors

**Dr Shannon Clarke** (Animal Genomics Science Team leader at AgResearch, New Zealand) has over 20 years’ experience in the development and implementation of genomic tools in the New Zealand livestock and aquaculture industries to enable validation and adoption of these technologies to drive genetic gain. These molecular tools range from low plex SNP marker systems for parentage and single gene traits to mid- to high-density SNP array- and sequencing-based methods suitable for a range of genomic applications. Aligned to development, validation and implementation of genotyping tools for industry are the research programs for functional annotation of the animal genomes, specifically the methylome and association with adaptation to the environment that she leads. Combining advances in technologies that will allow the genome architecture to be explored more efficiently, utilizing this knowledge and applying to selection for environmental adaptation will enable sustainable, productive farming to thrive in the changing global climate.

**Alex Caulton** is a final year PhD candidate at the University of Otago, in collaboration with AgResearch, New Zealand. In 2018, she graduated from the University of Otago with an MSc in genetics. Her masters project involved the development of a high-throughput genetic screen for production-relevant traits in farmed salmon and dairy goats, work that is now used in a commercial setting to facilitate breeding decisions. Her current research lies at the intersection of molecular genetics, epigenetics, bioinformatics, and the implementation of genomic tools within the livestock and aquaculture industries to drive genetic gain. She has an interest in functional annotation of animal genomes and has been involved in data collection, analysis, and “omics” data integration for the International Ovine FAANG project. She is also involved in biomarker development for age and health traits in livestock, specifically in the establishment of an epigenetic clock for New Zealand livestock species.

**Kathryn McRae** is a Senior Scientist in the Animal Genomics team at AgResearch. Her farming background, coupled with her interest in genetics, led her to complete a Master of Science in genetics with the University of Otago in collaboration with AgResearch. Subsequently, she graduated with a PhD from Dublin City University as a Teagasc Walsh Fellow. Both her MSc and PhD research investigated the genetic basis of resistance to gastrointestinal nematodes in sheep. Her scientific background includes molecular biology, genomics, genetics, parasitology, and bioinformatics. Her main research interest is using these skills to investigate the genetic basis of disease in livestock, including gastrointestinal nematode infection and respiratory disease. The goal of this research is to breed healthy, productive animals that are more resilient to external stressors.

**Rudiger Brauning** is a Senior Bioinformatician with AgResearch, New Zealand. He has a PhD in molecular genetics from the University of Heidelberg, Germany. His work is broadly focused on delivering genotyping solutions to the primary industry. With a cross-disciplinary team, he helped develop SNP arrays for sheep and establish Genotyping by Sequencing on a range of species. The emphasis of his work is on cost-effective, robust pipelines. He plays an active role in the International Sheep Genomics Consortium as well as advisory roles for training initiatives in the bioinformatics space.

**Christine Couldrey** is a Research Leader in Molecular Genetics at Livestock Improvement Corporation (LIC). Her journey in science began with a BSc (honors) in Biochemistry at Lincoln University (New Zealand), completing a PhD at Cambridge University (England) as a Commonwealth Scholar, and finally two postdoctoral research positions in the United States before returning to NZ to take up a (senior) scientist position at AgResearch. Her scientific training includes molecular biology, gene editing, genetics, epigenetics, and reproduction. These days, she combines her scientific skills to generate, analyze, and utilize DNA sequence data to identify disease-causing agents present in milk and feces and better understand the bovine genome. The ultimate goal of this work is to improve the health, well-being, milk production efficiency, and resilience of dairy cows in New Zealand.

**Ken Dodds** is a statistical geneticist at AgResearch, a New Zealand crown research institute. He has a PhD in statistics with minor in genetics from North Carolina State University. He uses statistical methods to help genetically improve and manage livestock and other species. He has developed and used methods to use genetic marker information including methods for genetic evaluation with fractionally assigned pedigrees and methods for using partial genotype information from low-depth sequencing data to inform plant and animal breeding, ecology, and conservation.
